# Proteomic Analysis Shows Synthetic Oleanane Triterpenoid Binds to mTOR

**DOI:** 10.1371/journal.pone.0022862

**Published:** 2011-07-27

**Authors:** Mark M. Yore, Arminja N. Kettenbach, Michael B. Sporn, Scott A. Gerber, Karen T. Liby

**Affiliations:** 1 Department of Pharmacology, Dartmouth Medical School, Hanover, New Hampshire, United States of America; 2 Department of Genetics, Dartmouth Medical School, Hanover, New Hampshire, United States of America; 3 Norris Cotton Cancer Center, Lebanon, New Hampshire, United States of America; Johns Hopkins University, United States of America

## Abstract

New multifunctional drugs that target multiple disease-relevant networks offer a novel approach to the prevention and treatment of many diseases. New synthetic oleanane triterpenoids (SO), such as CDDO (2-cyano-3,12-dioxooleana-1,9-dien-28-oic acid) and its derivatives, are multifunctional compounds originally developed for the prevention and treatment of inflammation and oxidative stress. However, the protein binding partners and mechanisms of action of these SO are not yet fully understood. Here we characterize the putative target profile of one SO, CDDO-Imidazolide (CDDO-Im), by combining affinity purification with mass spectroscopic proteomic analysis to identify 577 candidate binding proteins in whole cells. This SO pharmaco-interactome consists of a diverse but interconnected set of signaling networks; bioinformatic analysis of the protein interactome identified canonical signaling pathways targeted by the SO, including retinoic acid receptor (RAR), estrogen receptor (ER), insulin receptor (IR), janus kinase/signal transducers and activators of transcription (JAK/STAT), and phosphatase and tensin homolog (PTEN). Pull-down studies then further validated a subset of the putative targets. In addition, we now show for the first time that the mammalian target of rapamycin (mTOR) is a direct target of CDDO-Im. We also show that CDDO-Im blocks insulin-induced activation of this pathway by binding to mTOR and inhibiting its kinase activity. Our basic studies confirm that the SO, CDDO-Im, acts on a protein network to elicit its pharmacological activity.

## Introduction

Chronic diseases are now the prime cause of most pain and suffering in American society, as well as the largest financial burden in the health care system [1]. Their present treatment is often unsatisfactory. Multiple factors are now known to cause the initiation, as well as drive the progression, of chronic neurodegenerative, inflammatory, cardiovascular, metabolic, or neoplastic diseases [2]–[4]. Because of the intrinsic complexity of all of these diseases, we now need an innovative therapeutic approach that uses multifunctional pharmaceuticals to simultaneously target an array of pathogenetically relevant pathways, each of which contributes to disease progression [5]. Such “systems-based” therapeutics would have the marked advantage of subverting drug-resistance, improving efficacy, and reducing adverse side effects [5]–[6].

Over the past decade a series of synthetic triterpenoids based on the natural triterpenoid, oleanolic acid (OA) has been synthesized [7]–[9]. These synthetic oleanane triterpenoids (SO) are multifunctional and have antiproliferative [10]–[11], anti-angiogenic [12], anti-diabetic [13], pro-apoptotic [14]–[20], anti-inflammatory [10], [11], [21]–[23], and cytoprotective [24]–[25] activities. Furthermore, the SO have been successfully used to prevent and treat many chronic diseases in experimental animals, including cancer [10], [26]–[29], emphysema [30]–[31], macular degeneration [32], liver disease [33]–[34], neurodegenerative diseases [35]–[36] and nephrotoxicity [37] in both *in vitro* and *in vivo* models. The SO, CDDO-methyl ester (CDDO-Me, Bardoxolone methyl) is currently in late-stage clinical development for treatment of chronic kidney disease (diabetic nephropathy) [73].

In the development of second generation OA derivatives, two electrophilic Michael acceptor sites were incorporated in the A and C rings of OA to increase the reactivity of OA toward cellular targets [25], [38]. In cells SO mediate their potent pharmacological effects in part through interactions with cellular nucleophiles such as discrete, redox-sensitive sulfhydryl groups of cysteine (Cys) residues on proteins, via reversible Michael addition [7], [9], [25], [38]–[41]. Therefore, one may expect that SO should have many cellular protein binding partners, depending on cellular context and the nucleophilicity of specific cysteine residues in specific targets.

A few biologically important targets of SO have already been discovered. Activation of the KEAP1/Nrf2 pathway has been shown to be critical for many of the activities of both CDDO-Me and its close relative, CDDO-Imidazolide (CDDO-Im) [24], [25], [42]. However, the SO are known to modulate numerous pathways in cell culture and recently it was shown that many KEAP1/Nrf2-independent cell-signaling targets are also modulated following administration of CDDO-Im *in vivo* [43]. To date, no global study to identify the complete spectrum of binding partners of SO on a proteome-wide scale has been reported. Therefore, here we report the results of such a proteomics approach. We have utilized biotin tagged SO conjugates as chemical probes to affinity purify protein binding partners after treatment of cells in culture and have identified these proteins by LC-MS/MS. We report here the identification of 577 proteins to which a biotinylated probe binds. This approach also identified and validated binding partners which have previously been reported to be direct SO targets, including IKK, JAK1, PTEN and tubulin [39], [40], [44]–[45]. Our studies now show that the multi-functional SO, CDDO-Im, as expected, exerts its varied pharmacological actions by acting on large and diverse protein networks.

## Materials and Methods

### Reagents

CDDO-Im, TP-154 and TP-304 have been described previously [14], [46]–[50], TP-304 is Compound 6 in Ref. 49. They were dissolved in DMSO, and controls containing equal volumes of DMSO (<0.1%) were included in all experiments. Sources of commercial reagents were: rabbit polyclonal antibodies against HO-1, Keap-1, tubulin and GAPDH, Santa Cruz Biotechnology; all other antibodies were from Cell Signaling Technology. Insulin, LY294002, Rapamycin and all other chemicals were obtained from Sigma.

### Cell Culture

HEK293 and PC-3 cells were from ATCC, and maintained in DMEM (HEK293) or RPMI (PC-3), with 10% FBS and in 5% CO_2_. In some cases cells were plated and treated in culture media containing 1% horse serum. To measure proliferation, an MTT assay was performed as described previously [45].

### Cell Lysis, Western Blotting and mTORC1 Immunoprecipitation

For Western blots, HEK293 cells were washed twice with cold PBS and suspended in RIPA buffer (25 mM Tris-HCl pH 7.6, 150 mM NaCl, 1% NP-40, 1% sodium deoxycholate, 0.1% SDS, 1 mM sodium orthovanadate (Na_3_VO_4_), 1 mmol/L phenylmethylsulfonyl fluoride (PMSF), 10 µmol/L leupeptin, and 5 µg/ml aprotinin). Protein concentration in centrifuged lysates was determined by Bradford assay. Lysates were immunoblotted as described previously [39]. Blots were developed with either pico-supersignal or femto-supersignal (Fisher Scientific). For mTORC1 immunoprecipitation [51], cells were washed twice with PBS and suspended in cold lysis buffer B (10 mM KPO_4_, pH 7.2, 0.3% CHAPS (to preserve the mTOR-Raptor interaction), 1 mM EDTA, 5 mM EGTA, 10 mM MgCl_2_, 50 mM beta-glycerophosphate, 1 mM Na_3_VO_4_). Clarified lysate supernatants were incubated with anti-RAPTOR polyclonal antibodies for 2 h at 4°C, followed by incubation with protein A Sepharose beads for 2 h. Immunoprecipitates were washed three times in lysis buffer B, and resuspended in 50 µl of Laemmli loading buffer followed by immunoblotting with mTOR antibodies [52].

### Biotinylated-Triterpenoid target affinity purification and Gel Staining for Proteomics

HEK-293 cells were plated in DMEM with 1% horse serum. The following day cells were treated with biotinylated SO as indicated for 1 h. Cells were then washed twice with cold PBS and lysed in Tris-HCl, 1% Triton X-100, pH 7.4 (Buffer A). Centrifuged lysates were incubated with 50 µl of MyOne™ Streptavidin T1 paramagnetic Dynabeads® (Invitrogen Corporation, Carlsbad, CA) for 1 h. Dynabead®-biotinylated triterpenoid protein-complexes were isolated magnetically and washed a total of five times with buffer A. An additional sample was treated with TP-304 and washed with a more stringent wash buffer (100 mM TrisHCl pH 7.4, 150 mM NaCl, 1% (v/v) Triton X-100, 300 mM sodium actate, 0.1% (v/v) SDS) to disrupt protein complexes and remove non-specific binding proteins. Complexes were boiled in Laemmli loading buffer and separated on 4–20% gradient, SDS-PAGE gels followed by staining overnight in a solution of 50% MeOH, 40% H_2_O, 10% acetic acid and 0.125% Coomassie brilliant blue R-250.

### Sample preparation, MS and data anlysis

Coomassie-stained gels were divided into 8 regions and digested with trypsin overnight at room temperature. The next day, peptides were extracted, dried, and analyzed by nanoscale microcapillary LC-MS/MS as described. Samples were loaded using a FAMOS autosampler (LC Packings/Dionex, Sunnyvale CA) onto a custom manufactured reverse-phase, Reprosil-Pur 3 µm, 200 Å C_18_-AQ particle filled (Dr. Maisch GMBH, Ammerbuch-Entringen, Germany) fritless-tip microcapillary columns (0.125×180 mm, 7 µm tip) (Polymicro Technologies, Phoenix, AZ, and Sutter Instruments, Novato, CA) using an HPLC-driven (Agilent 1200 capLC) split-flow system designed to maintain 1200–1500 psi at the head of the analytical column. The resultant peptide eluate was directed into an LTQ-Orbitrap high performance mass spectrometer operating in a data-dependent sequencing acquisition mode across a 60-minute reverse-phase gradient (5% acetonitrile / 0.1% formic acid to 30% acetonitrile / 0.1% formic acid). The collected tandem mass spectra were data-searched using the SEQUEST algorithm [53], filtered to less than 1% false discovery rate using the target-decoy strategy [54] and reported.

### Pathway analysis

For Go annotation, UniProt IDs were submitted to GORetriever [55] and categorized in GO_slim categories using CateGOrizer [56]. Overall connectivity of identified proteins was determined using STRING [57]. Highly connective subnetworks in the STRING network were identified using MCODE plug-in into Cytoscape [58]. For network analysis, UniProt IDs were submitted to Ingenuity software (Ingenuity® Systems, www.Ingenuity.com).

## Results

### Functional validation of biotinylated SO conjugates confirms their use as chemical probes

**Figure 1:**
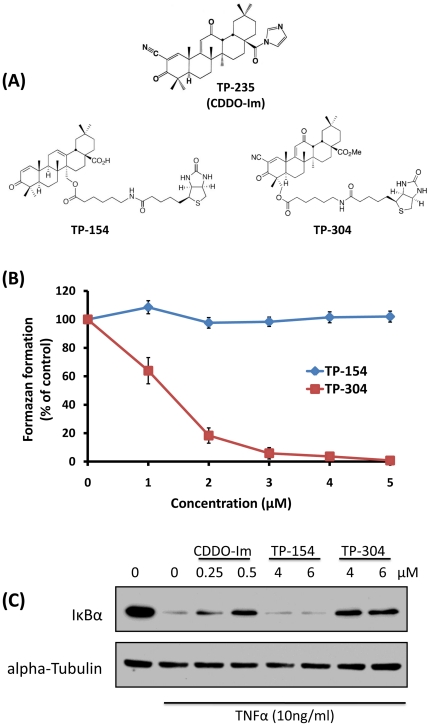
Characterization of SO for chemical proteomic studies. (A) The chemical structures of the SO, CDDO-Im (TP-235), TP-154 (inactive) and TP-304 (active). (B) TP-304 but not TP-154 inhibits the proliferation of HE293 cells. HEK293 cells were treated with either DMSO (control), TP-304 or TP-154 at the concentrations indicated for 48 h. Proliferation was assessed by MTT assay. (C) CDDO-Im and TP-304 but not TP-154 prevent the TNFα-induced degradation of IκBα. HEK293 cells were treated with DMSO (control), CDDO-Im, TP-304 or TP-154 at the concentrations indicated for 1 h followed by treatment with 10 ng/ml TNFα. Lysates were immunoblotted with antibodies against IκBα or tubulin (loading control).

To identify candidate cellular targets of SO, biotinylated SO (btSO) conjugates have been synthesized. The pharmacological activity of these btSO was initially measured here by their ability to inhibit cellular proliferation (Figures 1a, 1b). Biotinylated TP-304 strongly inhibited proliferation of HEK293 cells, while TP-154 was completely inactive in this assay (Figure 1b). Furthermore we compared TP-154 and TP-304 with a potent, non-biotinylated, SO, CDDO-Im. We examined their ability to inhibit the activation of the NFκB pathway as induced by TNFα, by measuring their prevention of the degradation of IκBα (Figure 1c). CDDO-Im, blocks IκBα degradation, indicating that it prevents activation of the NFκB pathway (Figure 1c). Again, these results are also seen with TP-304, whereas the inactive TP-154 fails to prevent IκBα degradation. Based on these results TP-304 and TP-154 were selected for use in subsequent proteomic studies. TP-304 was used as a chemical probe to affinity purify target proteins, whereas TP-154 was used as an inactive negative control.

### Proteomics analysis reveals multiple SO binding proteins

**Figure 2:**
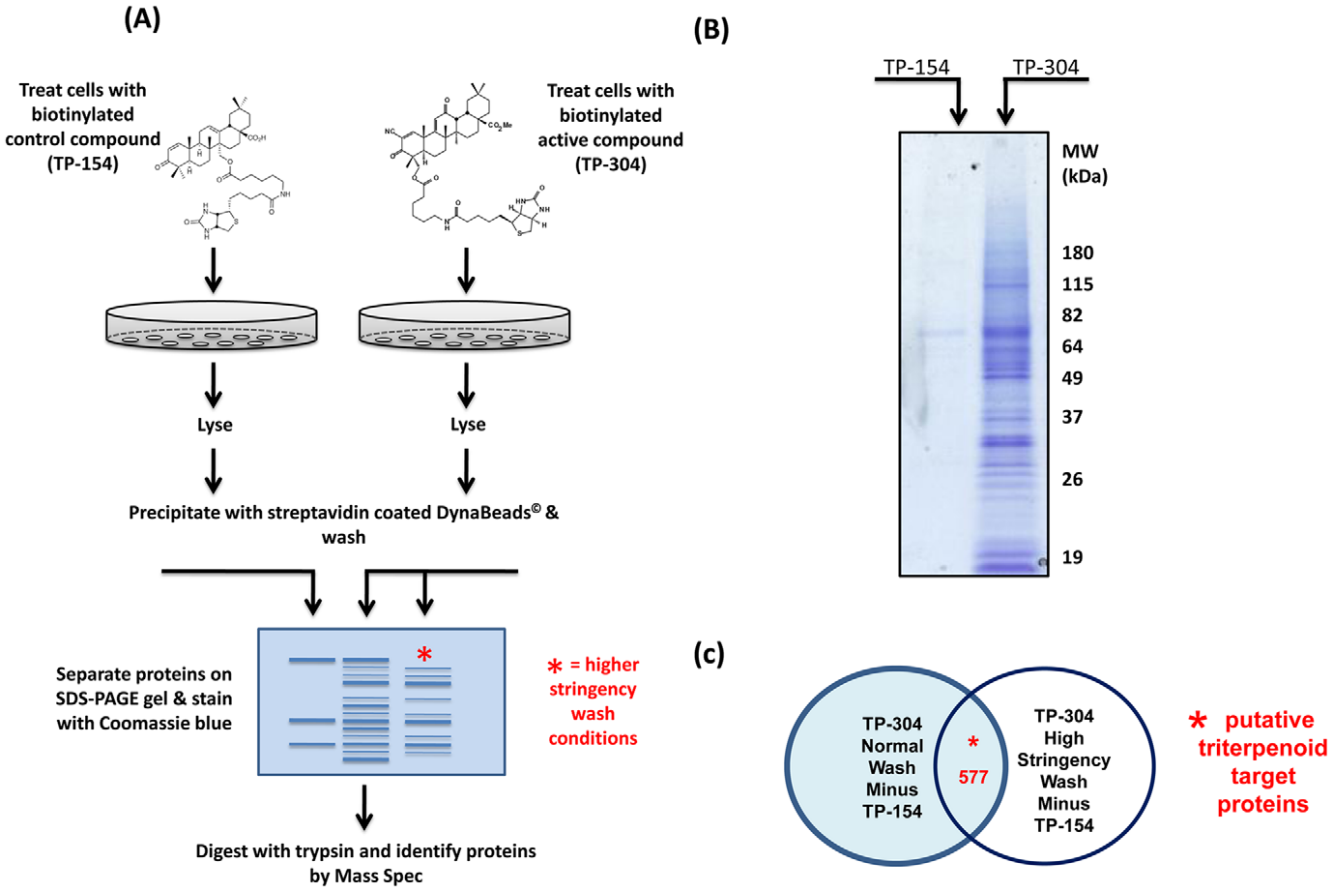
Chemical proteomics studies for identification of SO targets. (A) Schematic representation. HEK293 cells were treated with biotinylated SO compounds for 1 h in culture. Following cell lysis, target proteins were affinity purified with paramagnetic beads. Beads were washed extensively 5× and remaining proteins were eluted by boiling in Laemmli buffer. Proteins were separated by SDS-PAGE on 4–12% gradient gels. Gels were stained with Coomassie stain overnight followed by destaining until background staining was clear. Corresponding regions from both TP-154 and TP-304 gels were excised, trypsinised and protein identified by LC-MS/MS. (B) Image of Coomassie-stained gel submitted for mass-spectroscopic analysis. (C) Proteins identified in the TP-154 samples (control) were subtracted from the TP-304 treatment groups.

Utilizing TP-304 and TP-154 as probes, we developed and optimized protocols to treat cells in culture for proteomic analysis. Because of the electrophilic nature of the SO and their ability to bind to redox active targets, it was important to ensure that the biotinylated probes interact with protein binding partners in their natural environment where the context-dependent nucleophilicity of -SH groups in cysteine residues is preserved. Following treatment with TP-304 and TP-154, both at 4 µM, for 1 h, HEK293 cells were lysed, and lysates were incubated with Neutravidin-coated Dynabeads to affinity purify protein targets (Figure 2a). Affinity purified proteins were separated on SDS-PAGE gels and stained with Coomassie blue (Figure 2b). Bands were excised, trypsinized and proteins identified by LC-MS/MS. The collected tandem mass spectra were data-searched using the SEQUEST algorithm [53] to identify target proteins. Following MS detection, proteins found in the TP-154 control sample were subtracted from proteins that were common to TP-304-treated samples, in both the normal and increased stringency wash conditions. (Figure 2a, 2b & 2c). 577 proteins common to both the TP-304 normal wash and the TP-304 high stringency wash were subjected to bioinformatic analysis (Figure 2c). A complete list of binding proteins is detailed in Figure S1.

**Figure 3:**
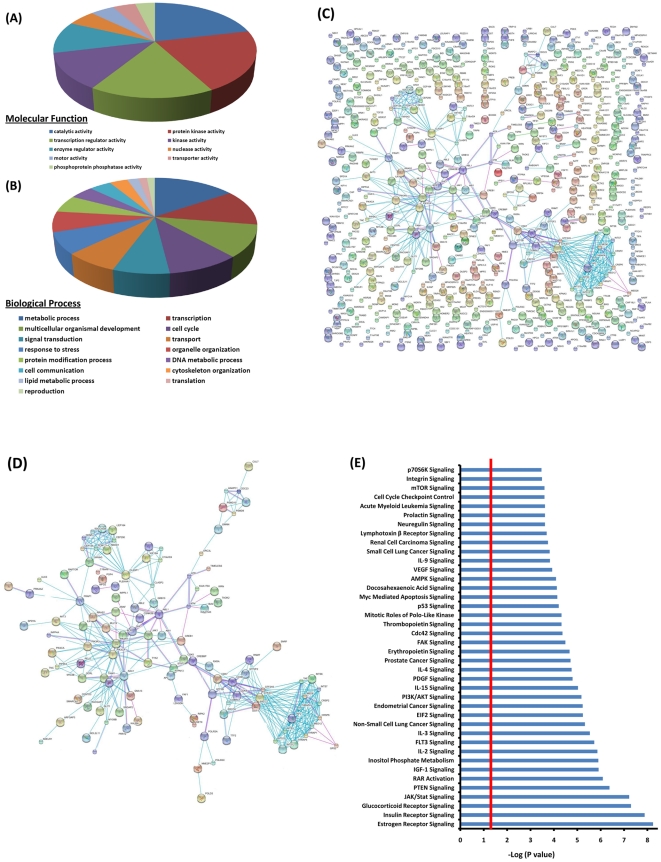
Bioinformatic analysis of putative SO target proteins. Gene ontology analysis of the candidate SO targets. GO_slim, gene ontology analysis of the candidate substrates was done with the CateGOrizer program. (**A**) SO target proteins, were annotated by molecular function. Categories with more than 20 assigned proteins are shown. (**B**) The 577 putative SO target proteins, were assigned to biological processes. Proteins for which no biological process could be assigned were omitted from this display. Categories with more than 20 assigned proteins are shown. (**C**) Overall connectivity of identified proteins was determined using STRING (Search Tool for the Retrieval of Interacting Genes/Proteins). (**D**) Highly connected sub-networks within the STRING network. Different line colors represent the types of evidence for each association, blue lines: direct binding, pink lines: post-translational modification. (**E**) For canonical pathway analysis, UniProt IDs were analyzed with Ingenuity pathway software. The top 40 most significant canonical signaling pathways from the Ingenuity canonical pathway library mapped to the SO target dataset are displayed. Threshold bar shows cut-off point of significance *P*<0.05, −log(*P*-value) of 1.3 as determined using a right-tailed Fisher's exact test. High resolution images of the STRING network and sub-network can be found in the supplementary information.

The putative SO targets were annotated using gene ontology (GO) identifiers. For GO annotation, UniProt IDs were submitted to GORetriever and categorized in GO_slim categories using CateGOrizer. GO annotation based on molecular function showed that catalytic activity, protein kinase activity, transcription regulator activity, kinase activity and enzyme regulator activity represented 75% of putative TP-304 targets (Figure 3a). GO categorization of SO targets by biological process identified metabolism, transcription, development, cell cycle, and signal transduction to be the most prominent biological processes targeted by TP-304 (Figure 3b). While TP-304 bound to a large number of proteins, these proteins form a highly interconnected network. This overall connectivity map (or interactome) was established using the STRING database of known and predicted protein interactions, including direct (physical) and indirect (functional) associations (Figure 3c and 3d; high resolution images Figures S2 and S3). Interestingly, analysis of the STRING, SO-protein interaction network identified a subset of densely connected regions representing molecular complexes of interconnected sub-networks (Figure 3d). This sub-network contained clusters enriched with proteins involved in cell-cycle/cell division, meiosis/mitosis. This is not surprising given the potent anti-proliferative activities of the SO. Interestingly, central to these enriched clusters of proteins were a number of proteins that represent common signal transduction pathways critical for cell-cycle and cell division including PI3K/AKT/mTOR, JAK/STAT and BCR-ABL (Figure 3d). These data correlate with the findings from the gene ontology molecular function analysis which highlighted proteins involved in catalytic, kinase and transcription regulatory functions as being central targets of the SO in biological processes that include metabolism, transcription, cell cycle and signal transduction (Figures 3a and 3b).

Thus we analyzed TP-304 targets using the Ingenuity software program to identify specific canonical pathways within the TP-304 binding partner dataset. This analysis identified a large number of canonical pathways targeted byTP-304 with very high significance (Figure 3e). The top 40 pathways are displayed in Figure 3e. While these analyses identified many new pathways with which CDDO-Im interacts, they also confirmed pathways for which the SO have previously been shown to modulate, including, janus kinase/signal transducers and activators of transcription (JAK/STAT), phosphatase and tensin homolog (PTEN), and AMP kinase (AMPK).

### Validation of a subset of putative SO target proteins

**Figure 4:**
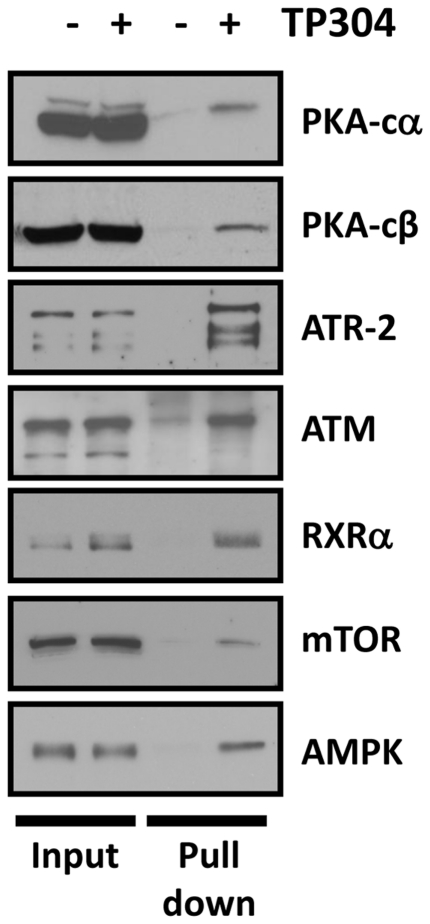
Validation of putative SO targets identified by LC-MS/MS. HE293 cells were treated with either DMSO (control) or 4 µM TP-304 for 1 h, as indicated. TP-304 binding proteins were affinity purified with NeutrAvidin resin. Proteins were separated by SDS-PAGE and immunoblots performed with various antibodies targeted toward a subset of proteins identified in the prior LC-MS/MS studies. Samples from whole cell lysate for both treatment conditions were also immunoblotted (lanes 1 and 2) to show that treatments had no effect on total protein levels.

Next we determined the validity of the putative SO binding partners identified in the above proteomics studies. We used methods previously employed to identify IKK as an SO target. HEK293 cells were treated with 4 µM TP-304 for 1 h, followed by lysis and pull-down of TP-304-protein binding partners with Neutravidin-coated agarose beads. Following a series of washes to eliminate nonspecific binding, the remaining bound proteins were eluted and separated by SDS-PAGE followed by Western blotting. This was performed for a subset of proteins specifically identified above (Figures 2c, 3) by LC-MS/MS. As shown in Figure 4, we found that TP-304 bound specifically to 10 proteins, including protein kinase A, catalytic unit alpha (PKA-Cα) and beta (PKA-Cβ), ataxia telangiectasia and rad3 related (ATR), ataxia telangiectasia mutated (ATM), retinoid X receptor α (RXR-α), mammalian target of rapamycin (mTOR), AMP-dependent protein kinase (AMPK) (Figure 4); and IκB-kinase (IKK), c-Jun, and STAT1 (data not shown). These results in Figure 4 validate the MS approach taken in Figures 2 and 3 above.

### Functional validation of putative SO targets confirms mTOR as a direct SO target

**Figure 5:**
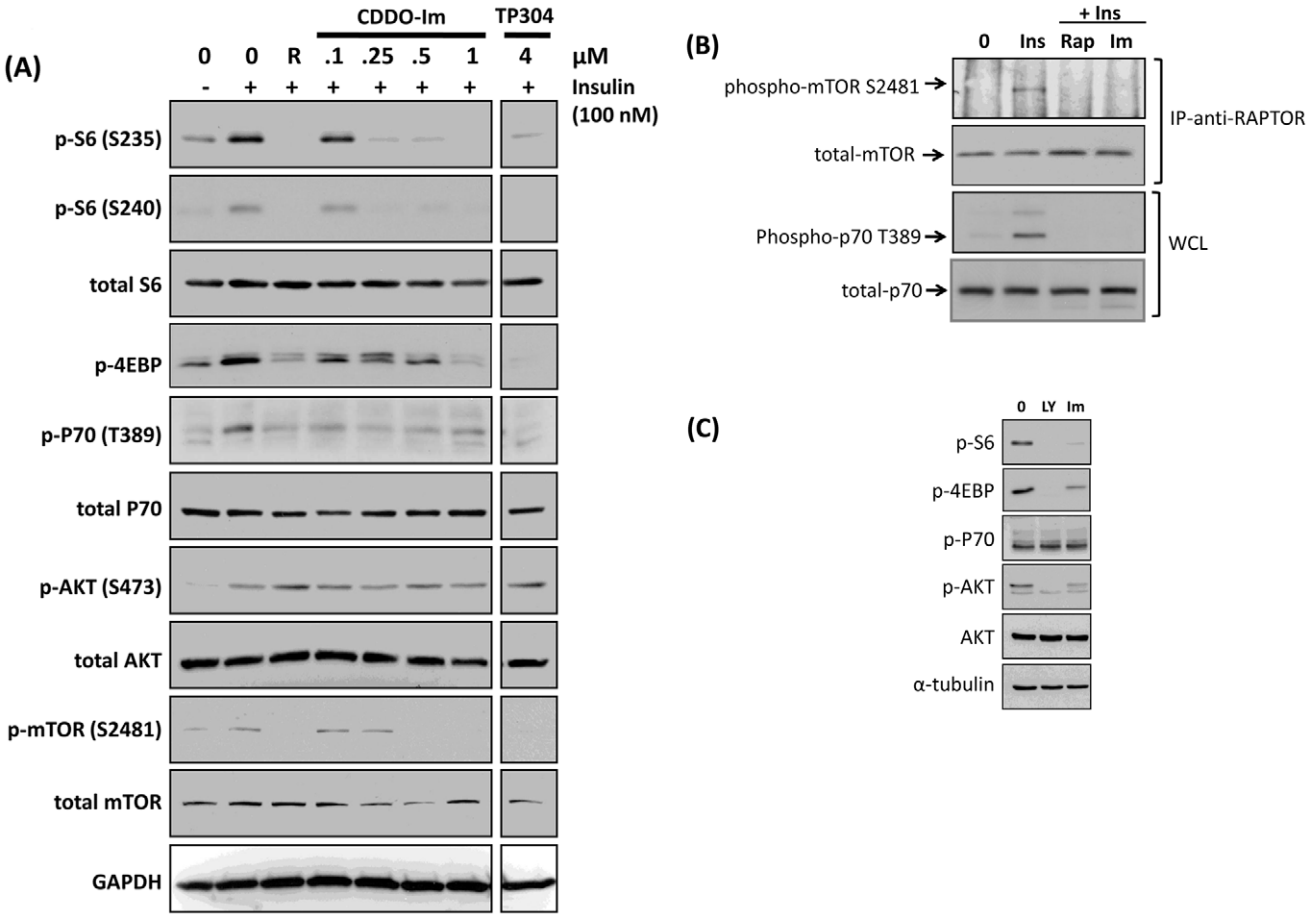
CDDO-Im inhibits the autokinase activity of mTOR. CDDO-Im inhibits the mTOR pathway in HEK293 cells. (A) HEK293 cells were treated with rapamycin (R, 20 nM) CDDO-Im or TP-304 as indicated for 1 h. Then, cells were treated with 100 nM Insulin for 30 min. Cells were lysed in RIPA buffer and Western immunoblotting was performed with the antibodies listed. (B) CDDO-Im inhibits the auto-kinase activity of mTOR. HEK293 cells were treated with DMSO, rapamycin (20 nM) or CDDO-Im (500 nM) for 2 h. Cells were then stimulated with 100 nM insulin for 15 min. Cells were lysed, and lysates were immunoprecipitated with anti-RAPTOR antibodies and protein A beads. Immunoprecipitates were separated by SDS-PAGE and membranes blotted with anti-phospho-mTOR serine 2481 antibodies (top panel). Membranes were stripped and re-probed with antibodies for total-mTOR (second panel). Whole cell lysates were Western blotted with antibodies against phospho-p70, threonine 389 (third panel) and alpha tubulin (bottom panel). (C) CDDO-Im inhibits the mTOR pathway in cancer cells. PC-3 cells were treated with 10 µM of the PI3K inhibitor, LY 294002 (LY) or, CDDO-Im (500 nM) for 1 h. Cells were lysed in RIPA buffer and Western immunoblotting was performed with the antibodies listed.

The SO can bind to nucleophilic Cys residues on binding partners through Michael addition. Therefore, we sought to confirm that such binding had a functional effect on some of the binding partners identified above. We interrogated the Ingenuity-assigned canonical pathways to identify specific SO binding partners which appear in multiple pathways, and reasoned that proteins that appear in multiple canonical pathways may represent critical SO target nodes. Thus, components of the PI3K/AKT/mTOR pathway were highly enriched in our canonical dataset. We used the highly potent CDDO analog, CDDO-Im for these studies and found that it was a potent inhibitor of the phosphorylation of insulin-stimulated ribosomal S6 (RS6) at serine 235/236 (S235/236). As can be seen in Figure 5A, this phosphorylation of S235/236 is dose-dependently inhibited by CDDO-Im with complete inhibition observed at 250 nM. Importantly, the biotinylated SO, TP-304 also inhibited the phosphorylation of RS6 S235/236 (data not shown). Similarly, a second phosphorylation site on S6, serine 240/244 (S240/244) was also inhibited by the CDDO-Im (Figure 5A and data not shown). Levels of total RS6 remained unchanged upon SO treatment. We observed similar inhibition of the translational repressor protein 4E-BP1 at serine 37. As expected, these effects on RS6 and 4E-BP1 were also observed upon treatment with the mTOR inhibitor, rapamycin. Both 4E-BP1 and RS6 are critically important regulators of mitogen-induced mRNA translation levels in cells and are downstream targets of the phosphatidylinositol-3-OH kinase (PI3K) signal-transduction cascade which also includes the kinases AKT, mTOR and P70S6K [59].

Thus, based on the observed enrichment for this pathway from our proteomics experiments and the observed inhibition of phosphorylation of both RS6 and 4E-BP1, we then investigated the effects of CDDO-Im on this pathway in greater detail in a hierarchical manner, beginning with RS6 and 4E-BP1 and working back up the pathway to determine at which level the CDDO-Im is inhibitory. Since P70S6 induces RS6 phosphorylation we looked at the insulin-induced activation of this important kinase. As expected, insulin rapidly induced phosphorylation of threonine 389 (T389) on P70S6K (Figure 5a). However, as observed with RS6 and 4E-BP1, CDDO-Im and rapamycin both prevented this activation, indicating that the inhibitory effects of the SO took place at a level above P70S6K activation. Since both P70S6K and 4E-BP1 are directly regulated by mTOR, and since mTOR and its binding partner, RAPTOR, were identified as direct targets for the SO in our proteomics screen we next examined whether the inhibitory effects of the SO on this pathway were mediated by disrupting the kinase activity of mTOR itself.

To examine the effects on mTOR in more detail we conducted an in-vitro mTOR activation assay which examines the phosphorylation levels of serine 2481 of mTOR [52]. Serine 2481 is an autophosphorylation site for this kinase [52]. mTORC1 was isolated by immunoprecipitating with anti-RAPTOR antibodies using a CHAPS-based lysis buffer which retains the integrity of the mTORC1 complex. Following immunoprecipitation, we immunoblotted with anti-mTOR S2481 antibodies to determine the level of phosphorylation at this autophosphorylation site. As can be seen in Figure 5b, in quiescent, serum-starved cells this site remains unphosphorylated but is rapidly phosphorylated (within 15 min) in response to insulin. This insulin-induced autophosphorylation is completely blocked by both rapamycin and CDDO-Im. Importantly, there was no effect on total mTOR levels immunoprecipitated by either rapamycin or CDDO-Im indicating that these treatments were not simply disrupting the mTOR-RAPTOR interaction. To further confirm the inhibition of mTOR in this experiment we performed Western blot experiments on whole cell lysates looking at phospho-P70S6K T389. As can be seen in Figure 5b, both rapamycin and CDDO-Im completely blocked autophosphorylation of P70S6K T389.

In order to exclude the possibility that the CDDO-Im was inactivating the pathway upstream of mTOR, and in turn blocking its activation we examined the activity of the upstream activating kinases, PI3K and AKT, by looking at phosphorylation levels of their substrates, AKT and GSK3β, respectively. As expected, we found that insulin rapidly phosphorylated AKT at S473 (Figure 5a). This effect was not blocked by either CDDO-Im or rapamycin, indicating that the CDDO-Im was not blocking activation of, or inhibiting PI3K, nor was it blocking activation of the mTOR complex 2 (mTORC2) which has recently been shown to phosphorylate AKT at S473. Again, these effects mimic that of rapamycin, which, under acute treatment conditions is unable to inhibit the activity of mTORC2. Furthermore, no effect was observed on phosphorylation of serine 9 of GSK3α/β (data not shown) indicating that activation or activity of AKT was not impaired by either CDDO-Im or Rapamycin (Figure 5a).

To determine if this inhibition of the mTOR signaling pathway was idiosyncratic to HEK293 cells we examined the effect of CDDO-Im in the prostate cell line, PC-3. PC-3 cells have constitutive activation of the PI3K/AKT/mTOR signaling axis due to mutations in the lipid phosphatase, PTEN, which dephosphorylates PI3K. In PC3 cells CDDO-Im inhibited the phosphorylation of RS6 S235/236 and 4E-BP1 S65 (Figure 5c), indicating the effects observed in the HEK293 cells were not unique to a single cell line.

## Discussion

Utilizing a novel, chemical proteomics approach, we have identified 577 proteins which interact with the biotinylated SO, TP-304, in cell culture. While some of these proteins had previously been identified, to date, this is the first report of a comprehensive and system-wide analysis of SO binding partners. As expected, a large number of new binding partners were identified, forming a large interconnected network of proteins that interact with SO. Many of the canonical pathways previously implicated as being targeted by SO, such as NFκB, JAK/STAT, PTEN and AMPK were found in this analysis [7], [32], [39], [40], [60]–[61].

In addition to these networks identified in silico, we have functionally validated the mTOR pathway as a target for CDDO-Im. Previous studies have suggested that this pathway may be inhibited by very high concentrations of the SO, CDDO-Me [62]–[63]. However, this is the first report to show that CDDO-Im also inhibits this pathway and also the first report that nanomolar concentrations of an SO directly inhibit the kinase activity of mTOR. mTOR belongs to a family of 6 kinases referred to as phosphoinositide-3-OH-kinase-related kinases (PIKKs). Interestingly the PIKK family members ATM and ATR and TRRAP were also pulled-down by TP-304.

The SO are extremely potent inhibitors of cellular proliferation [10]–[11]. The concentrations at which such inhibition is observed correlates with those we report for mTOR inhibition. Therefore, one may propose that this potent inhibition of proliferation may, in part, be due to inhibition of translation through disrupting mTOR activity. The identification of the PI3K/mTOR pathway as a central node in the STRING analysis interconnecting critical cell cycle and cell division proteins as well as the prominence of cell cycle proteins in the GO analysis would suggest this may be an important target for the SO. Also, it was recently reported that CDDO-Im alters microtubule dynamics by disrupting the microtubule-capping protein, Clip-170 which is an mTOR target protein [64]. Our observations suggest that the mechanism by which CDDO-Im disrupts Clip-170 may be through inhibition of the kinase activity of mTOR. Aberrant activation of mTOR and S6K has been shown to play a critical role in the development of diabetes and diabetic nephropathy [65]–[68]. Currently, CDDO-Me (Bardoxolone methyl) is undergoing late stage clinical development for the treatment of nephropathy in diabetes patients and significant improvements in markers of renal function have been reported [73]. Some of the improvements observed in these patients may be mediated through the inhibition of the mTOR/S6K signaling axis. Further studies toward this goal are ongoing in our laboratory.

One caveat of our proteomics-based target search is its qualitative nature, which lacks quantitative information about the SO-target interaction. Binding in such assays is a function of both protein/target abundance and affinity; therefore, highly abundant-low affinity proteins will co-purify with low abundance high-affinity proteins, making it difficult to infer relative affinities of the SO for the identified targets. However, multi-functional drugs typically bind to targets with lower affinity than a single target drug. Previous studies have suggested that low-affinity, multi-target drugs have a lower prevalence and a reduced range of side-effects than high-affinity, single-target drugs while also being more efficacious [7], [69]–[71]. By virtue of their low affinity binding, drugs of this type work best under pathological conditions, for the prevention of disease by restoring cellular homeostasis. Indeed it is in the area of chemoprevention that the SO have excelled in *in vivo* models. Currently the development of network-based, multi-target drugs similar in function to the SO is gaining increased interest in the field of drug development [7], [69]–[71]. Also, it should be noted that not all 577 proteins identified by MS analysis may be direct SO targets. It is possible that the lysis and wash conditions used did not completely disrupt high affinity protein complexes. We identified numerous proteins that are known to form high affinity complexes in cells including, but not limited to ATM/ATR and mTORC1. While this may cloud the identification of direct targets it facilitates the identification of protein complexes and pathways with which the SO interacts. One other caveat is the possibility that the coupling of the biotin group on the btSO may impede interactions with some target proteins. Indeed, TP-304 appears to have a lower affinity for KEAP1 (unpublished observation) which explains its absence from our list of proteins identified by MS. However, in spite of the above caveats, the data shown here open the way to new investigations that will examine the more difficult problem of interactions of SO with targets in real time.

In summary, technological advances in the integration of proteomics, pharmacology and molecular biology now provide the means to map drugs and targets at a systems level. Indeed, it has become evident that many drugs thought to specifically target a single protein, *e.g.* the BCR-ABL inhibitors imatinib, nilotinib, and dasatinib, are, in fact more promiscuous than originally thought [72]. Further advances in drug development, enhanced by an understanding of the genetic and epigenetic complexity of many chronic diseases, will come from drugs that target multiple components of critical signaling pathways and metabolic networks. In this regard, our studies demonstrate the utility of proteomic methods using biotinylated compounds as probes for identifying new candidate targets and perhaps may facilitate the use of SO in previously unrecognized therapeutic applications. The goals of targeting complex networks and applying systems biology to drug discovery are now of immediate importance.

## Supporting Information

Figure S1 Complete list of protein TP-304 binding proteins identified by LC-MS/MS. The collected tandem mass spectra were data-searched using the SEQUEST algorithm, filtered to less than 1% false discovery rate using the target-decoy strategy as described in the materials and methods section. The list is presented in descending order of total peptides (T.P.) and unique peptides (U.P.)
(PDF)

Figure S2 High resolution, scalable image of the network map depicted in figure 3c.
(TIF)

Figure S3 High resolution, scalable image of the sub-network map depicted in figure 3d.
(TIF)
