# Supplementary material for: Proteomic Analysis Shows Synthetic Oleanane Triterpenoid Binds to mTOR
Source: PLoS One. 2011 Jul 27;6(7):e22862. doi: 10.1371/journal.pone.0022862 (PMC3144948; doi:10.1371/journal.pone.0022862)
Supplement: Figure S1 — Complete list of protein TP-304 binding proteins identified by LC-MS/MS. The collected tandem mass spectra were data-searched using the SEQUEST algorithm, filtered to less than 1% false discovery rate using the target-decoy strategy as described in the materials and methods section. The list is presented in descending order of total peptides (T.P.) and unique peptides (U.P.) (PDF) [file pone.0022862.s001.pdf]

reference	TP	UP
Q9NU22|MDN1_HUMAN	56	40
Q9Y4A5|TRRAP_HUMAN	30	29
Q9NZJ4|SACS_HUMAN	34	28
Q9NZM3|ITSN2_HUMAN	39	25
P24821|TENA_HUMAN	36	24
Q13535|ATR_HUMAN	24	23
O60293|CC131_HUMAN	24	23
Q7Z460|CLAP1_HUMAN	31	22
O14578|CTRO_HUMAN	24	22
Q6VY07|PACS1_HUMAN	28	21
Q14966|ZN638_HUMAN	29	20
Q5VU43|MYOME_HUMAN	24	20
O95425|SVIL_HUMAN	22	20
P42336|PK3CA_HUMAN	44	19
Q13315|ATM_HUMAN	19	19
Q9NQW6|ANLN_HUMAN	19	18
Q9HD67|MYO10_HUMAN	18	17
O75691|UTP20_HUMAN	18	17
O15078|CE290_HUMAN	17	17
Q12923|PTN13_HUMAN	18	16
O94833|BPAEA_HUMAN	18	16
Q13796|SHRM2_HUMAN	17	14
Q9NVI1|FANCI_HUMAN	15	14
Q86V48|LUZP1_HUMAN	14	14
Q03468|ERCC6_HUMAN	14	14
Q15058|KIF14_HUMAN	18	13
Q93074|MED12_HUMAN	16	13
O94822|ZN294_HUMAN	15	13
Q8N201|INT1_HUMAN	13	13
Q86WG5|MTMRD_HUMAN	13	13
P46013|KI67_HUMAN	19	12
Q5T6F2|UBAP2_HUMAN	17	12
Q8N122|RPTOR_HUMAN	15	12
Q14999|CUL7_HUMAN	13	12
Q13459|MYO9B_HUMAN	13	12
Q9C0D5|TANC1_HUMAN	13	12
Q14674|ESPL1_HUMAN	12	12
Q9UPN3|MACF1_HUMAN	12	12
Q5T5Y3|CAMP1_HUMAN	12	12
O95714|HERC2_HUMAN	12	12
P27986|P85A_HUMAN	16	11
P42345|FRAP_HUMAN	13	11
Q9UPT8|ZC3H4_HUMAN	13	11
P33981|TTK_HUMAN	13	11
Q14692|BMS1_HUMAN	13	11

O60879|DIAP2_HUMAN	13	11
Q7Z6E9|RBBP6_HUMAN	13	11
Q96Q15|SMG1_HUMAN	12	11
A3KMH1|A3KMH1_HUMAN	11	11
O60313|OPA1_HUMAN	11	11
Q13009|TIAM1_HUMAN	11	11
Q7Z401|MYCPP_HUMAN	11	11
P78332|RBM6_HUMAN	11	10
Q92576|PHF3_HUMAN	10	10
P42356|PI4KA_HUMAN	10	10
Q8IY18|SMC5_HUMAN	10	10
Q9Y613|FHOD1_HUMAN	10	10
Q92793|CBP_HUMAN	10	10
Q9BY12|SCAPE_HUMAN	10	10
Q9Y2I8|WDR37_HUMAN	21	9
Q9GZR7|DDX24_HUMAN	13	9
Q9H7N4|SFR19_HUMAN	12	9
O75330|HMMR_HUMAN	10	9
Q96JC1|VPS39_HUMAN	10	9
Q9H425|CA198_HUMAN	10	9
O60244|MED14_HUMAN	10	9
P29597|TYK2_HUMAN	10	9
O60318|MCM3A_HUMAN	9	9
Q9UG01|IF172_HUMAN	9	9
Q9P2K8|E2AK4_HUMAN	9	9
Q9H9P8|L2HDH_HUMAN	10	8
Q5VW36|K1797_HUMAN	9	8
Q969X6|CIR1A_HUMAN	8	8
Q13322|GRB10_HUMAN	8	8
Q8WVX9|FACR1_HUMAN	8	8
Q86US8|EST1A_HUMAN	8	8
Q14137|BOP1_HUMAN	8	8
Q9UK41|VPS28_HUMAN	8	8
Q9BQ67|GRWD1_HUMAN	12	7
Q96S66|CLCC1_HUMAN	11	7
Q8TED0|UTP15_HUMAN	10	7
Q96RS6|NUDC1_HUMAN	10	7
Q14168|MPP2_HUMAN	10	7
O43148|MCES_HUMAN	10	7
P53804|TTC3_HUMAN	9	7
Q9Y3C6|PPIL1_HUMAN	8	7
Q6P158|DHX57_HUMAN	8	7
Q9H270|VPS11_HUMAN	8	7
Q6P1X5|TAF2_HUMAN	8	7
Q9HAU0|PKHA5_HUMAN	8	7
Q5TCQ9|MAGI3_HUMAN	8	7
Q9NRR4|RNC_HUMAN	8	7

Q8TCG1|K1524_HUMAN	8	7
Q8N3X1|FNBP4_HUMAN	7	7
Q7Z333|SETX_HUMAN	7	7
Q68CZ6|CD015_HUMAN	7	7
Q6PCB5|RSBNL_HUMAN	7	7
Q8WVM7|STAG1_HUMAN	7	7
Q92547|TOPB1_HUMAN	7	7
Q5H9R5|Q5H9R5_HUMAN	7	7
Q9UPP1|PHF8_HUMAN	7	7
B0I1T5|B0I1T5_HUMAN	7	7
Q15645|TRP13_HUMAN	7	7
Q9P2N5|RBM27_HUMAN	7	7
Q7Z5K2|WAPL_HUMAN	7	7
O75449|KTNA1_HUMAN	7	7
Q9Y450|HBS1L_HUMAN	12	6
Q06787|FMR1_HUMAN	11	6
Q14739|LBR_HUMAN	10	6
Q71RC2|LARP4_HUMAN	10	6
Q15061|WDR43_HUMAN	9	6
Q5T3I0|GPTC4_HUMAN	8	6
Q9NQT5|EXOS3_HUMAN	8	6
P82663|RT25_HUMAN	8	6
Q9UPQ6|Q9UPQ6_HUMAN	8	6
Q00535|CDK5_HUMAN	8	6
Q6KC79|NIPBL_HUMAN	8	6
O94885|SASH1_HUMAN	7	6
P22830|HEMH_HUMAN	7	6
Q8IYS1|P20D2_HUMAN	7	6
Q9Y263|PLAP_HUMAN	7	6
Q9H0C8|ILKAP_HUMAN	7	6
O43447|PPIH_HUMAN	7	6
P23458|JAK1_HUMAN	7	6
Q96EV2|RBM33_HUMAN	7	6
O75131|CPNE3_HUMAN	7	6
Q92794|MYST3_HUMAN	7	6
Q96D53|ADCK4_HUMAN	6	6
Q9BVA0|KTNB1_HUMAN	6	6
A0FGR8|ESYT2_HUMAN	6	6
Q5JPH6|SYEM_HUMAN	6	6
A6NK10|A6NK10_HUMAN	6	6
Q9NYY8|FAKD2_HUMAN	6	6
O75970|MPDZ_HUMAN	6	6
Q9H269|VPS16_HUMAN	6	6
Q96L91|EP400_HUMAN	6	6
Q9H4A3|WNK1_HUMAN	6	6
Q9Y4B5|K0802_HUMAN	6	6
O43314|VIP2_HUMAN	6	6

Q9GZR2|REXO4_HUMAN	6	6
O14802|RPC1_HUMAN	6	6
Q9P289|MST4_HUMAN	6	6
Q9BRX2|PELO_HUMAN	6	6
Q15650|TRIP4_HUMAN	6	6
Q9Y5S2|MRCKB_HUMAN	6	6
Q5VTL8|PR38B_HUMAN	10	5
Q9Y5J1|UTP18_HUMAN	7	5
Q9HCD6|TANC2_HUMAN	7	5
Q9BTC0|DIDO1_HUMAN	7	5
Q66K74|MAP1S_HUMAN	7	5
O14874|BCKD_HUMAN	6	5
B2RXK3|B2RXK3_HUMAN	6	5
P28288|ABCD3_HUMAN	6	5
Q9P2D3|HTR5B_HUMAN	6	5
Q9BW19|KIFC1_HUMAN	6	5
P50613|CDK7_HUMAN	6	5
Q9UGV2|NDRG3_HUMAN	6	5
Q9NX40|OCAD1_HUMAN	6	5
O75127|PTCD1_HUMAN	6	5
P51531|SMCA2_HUMAN	6	5
Q6ZNB6|NFXL1_HUMAN	6	5
Q8IWA0|WDR75_HUMAN	6	5
P43490|NAMPT_HUMAN	5	5
Q9Y3I1|FBX7_HUMAN	5	5
P56182|RRP1_HUMAN	5	5
O75665|OFD1_HUMAN	5	5
P43897|EFTS_HUMAN	5	5
Q9NV70|EXOC1_HUMAN	5	5
Q2T9J0|TYSD1_HUMAN	5	5
Q92545|TM131_HUMAN	5	5
Q8IWP9|CC28A_HUMAN	5	5
Q9NUL3|STAU2_HUMAN	5	5
O14981|BTAF1_HUMAN	5	5
Q8IX90|CM003_HUMAN	5	5
Q9UJX2|CDC23_HUMAN	5	5
Q9NXV6|CARF_HUMAN	5	5
O75448|MED24_HUMAN	5	5
Q8ND04|CQ071_HUMAN	5	5
Q9BZG1|RAB34_HUMAN	5	5
Q86YS7|K0528_HUMAN	5	5
Q5T7W0|ZN618_HUMAN	5	5
Q13627|DYR1A_HUMAN	5	5
Q96A72|MGN2_HUMAN	5	5
O94874|K0776_HUMAN	5	5
Q9Y4P3|TBL2_HUMAN	5	5
Q9Y2R4|DDX52_HUMAN	8	4

O96006|ZBED1_HUMAN	8	4
Q8WX92|NELFB_HUMAN	7	4
Q9H8H2|DDX31_HUMAN	7	4
P29353|SHC1_HUMAN	6	4
Q92564|DCNL4_HUMAN	6	4
O14646|CHD1_HUMAN	6	4
O60287|NPA1P_HUMAN	6	4
Q9HA92|RSAD1_HUMAN	5	4
Q8TDX7|NEK7_HUMAN	5	4
Q7Z3U7|MON2_HUMAN	5	4
Q9UBC2|EP15R_HUMAN	5	4
Q9UPV0|CE164_HUMAN	5	4
Q68E01|INT3_HUMAN	5	4
Q96PE3|INP4A_HUMAN	5	4
P68431|H31_HUMAN	5	4
Q9NVH2|INT7_HUMAN	5	4
Q9P1Y5|K1543_HUMAN	4	4
O95235|KI20A_HUMAN	4	4
Q1ED39|TS118_HUMAN	4	4
Q9H7E9|CH033_HUMAN	4	4
Q99653|CHP1_HUMAN	4	4
P09543|CN37_HUMAN	4	4
Q5VZL5|ZMYM4_HUMAN	4	4
P20585|MSH3_HUMAN	4	4
Q9NUD5|ZCHC3_HUMAN	4	4
Q15050|RRS1_HUMAN	4	4
O43166|SI1L1_HUMAN	4	4
P09012|SNRPA_HUMAN	4	4
Q96RE7|BTB14_HUMAN	4	4
Q6P3X3|TTC27_HUMAN	4	4
Q9P253|VPS18_HUMAN	4	4
Q9UL40|ZN346_HUMAN	4	4
Q92947|GCDH_HUMAN	4	4
Q9UBW7|ZMYM2_HUMAN	4	4
Q15154|PCM1_HUMAN	4	4
Q7L8L6|FAKD5_HUMAN	4	4
O60229|KALRN_HUMAN	4	4
Q5C9Z4|NOM1_HUMAN	4	4
P50748|KNTC1_HUMAN	4	4
Q86W92|LIPB1_HUMAN	4	4
P42566|EP15_HUMAN	4	4
P62140|PP1B_HUMAN	4	4
Q6P1K8|T2H2L_HUMAN	4	4
P50750|CDK9_HUMAN	4	4
P37268|FDFT_HUMAN	4	4
Q9UNN5|FAF1_HUMAN	4	4
Q66GS9|CP135_HUMAN	4	4

O00566|MPP10_HUMAN	4	4
Q8WX93|PALLD_HUMAN	4	4
Q86VP3|PACS2_HUMAN	4	4
Q96BD8|SKA1_HUMAN	4	4
Q96JN8|K1787_HUMAN	4	4
Q8WUY1|CH055_HUMAN	4	4
Q9H0P0|5NT3_HUMAN	4	4
Q9UNS1|TIM_HUMAN	4	4
O94964|CT117_HUMAN	4	4
Q8WV22|NSE1_HUMAN	4	4
P61011|SRP54_HUMAN	4	4
Q9H4I3|TRABD_HUMAN	4	4
Q9HC21|DNC_HUMAN	4	4
Q7Z739|YTHD3_HUMAN	4	4
O60292|SI1L3_HUMAN	4	4
Q9HCJ0|TNR6C_HUMAN	4	4
P00519|ABL1_HUMAN	4	4
Q13418|ILK_HUMAN	4	4
Q9H1I8|ASCC2_HUMAN	4	4
A8MQ02|A8MQ02_HUMAN	5	3
Q86VV8|RTTN_HUMAN	4	3
A8K1V8|A8K1V8_HUMAN	4	3
Q9Y4X0|AMER1_HUMAN	4	3
O60830|TI17B_HUMAN	4	3
Q96GA3|LTV1_HUMAN	4	3
P37235|HPCL1_HUMAN	4	3
Q9Y5X2|SNX8_HUMAN	4	3
Q9Y2Z4|SYYM_HUMAN	4	3
O15075|DCLK1_HUMAN	4	3
O60361|NDK8_HUMAN	4	3
O75044|FNBP2_HUMAN	4	3
Q9Y6X3|K0892_HUMAN	4	3
P54132|BLM_HUMAN	4	3
O95707|RPP29_HUMAN	4	3
Q00536|PCTK1_HUMAN	4	3
Q9BT17|MTG1_HUMAN	3	3
Q9UBI9|HDC_HUMAN	3	3
O43149|ZZEF1_HUMAN	3	3
Q9UGP4|LIMD1_HUMAN	3	3
O43681|ARSA1_HUMAN	3	3
P46100|ATRX_HUMAN	3	3
Q9H3S7|PTN23_HUMAN	3	3
Q9NY12|NOLA1_HUMAN	3	3
Q14191|WRN_HUMAN	3	3
Q8ND83|SLAI1_HUMAN	3	3
P51946|CCNH_HUMAN	3	3
O43521|BIM_HUMAN	3	3

Q96MF7|NSE2_HUMAN	3	3
Q9UPS8|ANR26_HUMAN	3	3
O00471|EXOC5_HUMAN	3	3
Q9HC77|CENPJ_HUMAN	3	3
Q9Y4F3|LKAP_HUMAN	3	3
Q9Y5T5|UBP16_HUMAN	3	3
P11274|BCR_HUMAN	3	3
Q9Y697|NFS1_HUMAN	3	3
Q5W189|Q5W189_HUMAN	3	3
Q9BZ29|DOCK9_HUMAN	3	3
Q9NXK8|FXL12_HUMAN	3	3
Q5SW96|ARH_HUMAN	3	3
Q96PY6|NEK1_HUMAN	3	3
Q13601|KRR1_HUMAN	3	3
Q9NY93|DDX56_HUMAN	3	3
P30044|PRDX5_HUMAN	3	3
O43583|DENR_HUMAN	3	3
Q96AX1|VP33A_HUMAN	3	3
Q9BVI4|NOC4L_HUMAN	3	3
Q9H0B6|KLC2_HUMAN	3	3
O75832|PSD10_HUMAN	3	3
Q8NFP7|NUD10_HUMAN	3	3
Q5T4S7|UBR4_HUMAN	3	3
Q86XI2|CNDG2_HUMAN	3	3
O75586|MED6_HUMAN	3	3
Q5PRF9|SAM4B_HUMAN	3	3
Q5TB80|QN1_HUMAN	3	3
O15446|RPA34_HUMAN	3	3
Q86YD1|PTOV1_HUMAN	3	3
Q9UKT4|FBX5_HUMAN	3	3
Q86SQ7|SDCG8_HUMAN	3	3
Q9BXS6|NUSAP_HUMAN	3	3
Q8TF46|DI3L1_HUMAN	3	3
Q01484|ANK2_HUMAN	3	3
Q96Q89|MPPH1_HUMAN	3	3
Q70E73|RAPH1_HUMAN	3	3
Q9BX40|LS14B_HUMAN	3	3
P49454|CENPF_HUMAN	3	3
Q9BQI0|IBA2_HUMAN	3	3
Q9BTA9|WAC_HUMAN	3	3
O43741|AAKB2_HUMAN	3	3
Q9UL54|TAOK2_HUMAN	3	3
Q6PD62|CTR9_HUMAN	3	3
O75496|GEMI_HUMAN	3	3
Q8NCM8|DYHC2_HUMAN	3	3
Q9HCU5|PREB_HUMAN	3	3
O15344|TRI18_HUMAN	3	3

P42684|ABL2_HUMAN	3	3
O43824|GTPB6_HUMAN	3	3
Q9H4B6|SAV1_HUMAN	3	3
Q7Z6M4|MTER2_HUMAN	3	3
Q8WVV9|HNRLL_HUMAN	3	3
Q6P4I2|WDR73_HUMAN	3	3
Q00765|REEP5_HUMAN	3	3
Q8TBP6|S2540_HUMAN	3	3
Q9ULE0|WWC3_HUMAN	3	3
Q9H0H5|RGAP1_HUMAN	3	3
Q6ZU35|K1211_HUMAN	3	3
P19793|RXRA_HUMAN	3	3
Q9UKJ3|GPTC8_HUMAN	3	3
O00233|PSMD9_HUMAN	3	3
O43617|TPPC3_HUMAN	3	3
Q69YN2|C19L1_HUMAN	3	3
Q7Z569|BRAP_HUMAN	3	3
Q8NHV4|NEDD1_HUMAN	3	3
Q15005|SPCS2_HUMAN	3	3
O15357|SHIP2_HUMAN	3	3
Q9UHV7|MED13_HUMAN	3	3
Q9H1A4|APC1_HUMAN	3	3
P63000|RAC1_HUMAN	3	3
Q49A26|NP60_HUMAN	3	3
P52789|HXK2_HUMAN	3	3
Q5T280|CI114_HUMAN	3	3
Q86X10|K1219_HUMAN	3	3
Q9Y620|RA54B_HUMAN	3	3
Q9UJX3|APC7_HUMAN	3	3
Q9BRV8|SIKE_HUMAN	3	3
Q99961|SH3G1_HUMAN	3	3
O43663|PRC1_HUMAN	3	3
Q15811|ITSN1_HUMAN	3	3
Q6P5Z2|PKN3_HUMAN	3	3
Q14344|GNA13_HUMAN	3	3
Q9Y448|T4AF1_HUMAN	3	3
Q9GZS1|RPA49_HUMAN	3	3
Q8N9N2|ASCC1_HUMAN	3	3
P32780|TF2H1_HUMAN	3	3
Q9NWS0|PIHD1_HUMAN	3	3
Q9Y2X0|MED16_HUMAN	3	3
Q15120|PDK3_HUMAN	3	3
Q8WUD4|CCD12_HUMAN	3	3
Q6PHR2|ULK3_HUMAN	3	3
P67870|CSK2B_HUMAN	3	3
Q9BVQ7|SPA5L_HUMAN	5	2
Q8WWK9|CKAP2_HUMAN	4	2

P24390|ERD21_HUMAN	4	2
Q9NZN8|CNOT2_HUMAN	4	2
Q8NCE2|MTMRE_HUMAN	4	2
Q9BUI4|RPC3_HUMAN	3	2
Q3SXM5|HSDL1_HUMAN	3	2
P10155|RO60_HUMAN	3	2
P41219|PERI_HUMAN	3	2
Q9NY61|AATF_HUMAN	3	2
Q96H35|RBM18_HUMAN	3	2
Q9Y6B7|AP4B1_HUMAN	3	2
Q15633|TRBP2_HUMAN	3	2
O96007|MOS2L_HUMAN	3	2
Q14568|HS902_HUMAN	3	2
B1AN89|B1AN89_HUMAN	3	2
Q9H061|T126A_HUMAN	3	2
Q8TDM6|DLG5_HUMAN	3	2
Q6UXV4|APOOL_HUMAN	3	2
Q9NR28|DBLOH_HUMAN	3	2
P52306|GDS1_HUMAN	3	2
Q66PJ3|AR6P4_HUMAN	3	2
Q9H079|CO029_HUMAN	3	2
B2RTR1|B2RTR1_HUMAN	3	2
Q06587|RING1_HUMAN	3	2
P61020|RAB5B_HUMAN	3	2
Q9NYR9|KBRS2_HUMAN	2	2
O75648|TRMU_HUMAN	2	2
Q00537|PCTK2_HUMAN	2	2
Q9NPI6|DCP1A_HUMAN	2	2
Q9BXK1|KLF16_HUMAN	2	2
Q8IXM2|CQ049_HUMAN	2	2
B2RWN5|B2RWN5_HUMAN	2	2
Q9BVV7|TI21L_HUMAN	2	2
Q15545|TAF7_HUMAN	2	2
Q9NSK0|KLC4_HUMAN	2	2
Q8IWC1|MA7D3_HUMAN	2	2
P61981|1433G_HUMAN	2	2
Q9NX08|COMD8_HUMAN	2	2
Q53H47|SETMR_HUMAN	2	2
Q01968|OCRL_HUMAN	2	2
Q9BWL3|CA043_HUMAN	2	2
P55957|BID_HUMAN	2	2
Q15654|TRIP6_HUMAN	2	2
Q6IBW4|CNDH2_HUMAN	2	2
Q7Z2Z1|CO042_HUMAN	2	2
P36969|GPX4_HUMAN	2	2
Q6P9H4|CNKR3_HUMAN	2	2
P25054|APC_HUMAN	2	2

Q9Y3P9|RBGP1_HUMAN	2	2
Q12980|CP035_HUMAN	2	2
P62070|RRAS2_HUMAN	2	2
Q9UNY4|TTF2_HUMAN	2	2
Q99661|KIF2C_HUMAN	2	2
Q53H80|CF166_HUMAN	2	2
Q9HB21|PKHA1_HUMAN	2	2
Q8NI77|KI18A_HUMAN	2	2
P14649|MYL6B_HUMAN	2	2
Q9NR56|MBNL1_HUMAN	2	2
Q8WVJ2|NUDC2_HUMAN	2	2
Q15678|PTN14_HUMAN	2	2
Q9H204|MED28_HUMAN	2	2
P49406|RM19_HUMAN	2	2
P10398|ARAF_HUMAN	2	2
Q15054|DPOD3_HUMAN	2	2
Q9NS69|TOM22_HUMAN	2	2
Q8WWN8|CEND3_HUMAN	2	2
Q6XZF7|DNMBP_HUMAN	2	2
Q13889|TF2H3_HUMAN	2	2
Q96EF6|FBX17_HUMAN	2	2
Q9BY89|K1671_HUMAN	2	2
Q8IWJ2|GCC2_HUMAN	2	2
Q8NDF8|PAPD5_HUMAN	2	2
Q9BUK6|MSTO1_HUMAN	2	2
Q7Z6J8|UB2CB_HUMAN	2	2
Q9NP61|ARFG3_HUMAN	2	2
O15439|MRP4_HUMAN	2	2
Q99470|SDF2_HUMAN	2	2
Q9H213|MAGH1_HUMAN	2	2
P54646|AAPK2_HUMAN	2	2
Q68D91|YE006_HUMAN	2	2
Q9BZL4|PP12C_HUMAN	2	2
A8MWQ3|A8MWQ3_HUMAN	2	2
Q8IY17|PLPL6_HUMAN	2	2
Q9NWU2|CT011_HUMAN	2	2
Q14CB8|RHG19_HUMAN	2	2
P13984|T2FB_HUMAN	2	2
Q9UHQ1|NARF_HUMAN	2	2
Q9ULL5|PRR12_HUMAN	2	2
Q8IZ69|HTF9C_HUMAN	2	2
Q00013|EM55_HUMAN	2	2
Q9BUL8|PDC10_HUMAN	2	2
O95057|DIRA1_HUMAN	2	2
Q9UFW8|CGBP1_HUMAN	2	2
Q9NRA8|4ET_HUMAN	2	2
Q9H000|MKRN2_HUMAN	2	2

O00505|IMA3_HUMAN	2	2
Q96AY4|TTC28_HUMAN	2	2
P52630|STAT2_HUMAN	2	2
Q9HCD5|NCOA5_HUMAN	2	2
O95429|BAG4_HUMAN	2	2
Q13232|NDK3_HUMAN	2	2
Q9NWU5|RM22_HUMAN	2	2
Q96C19|EFHD2_HUMAN	2	2
Q86XL3|ANKL2_HUMAN	2	2
Q8WYQ5|DGCR8_HUMAN	2	2
Q9H0W8|CS061_HUMAN	2	2
Q9Y2I7|FYV1_HUMAN	2	2
Q9H019|FA54B_HUMAN	2	2
O60831|PRAF2_HUMAN	2	2
Q5EBL8|PDZ11_HUMAN	2	2
Q0P651|CD029_HUMAN	2	2
Q9NSB2|KRT84_HUMAN	2	2
Q9C035|TRIM5_HUMAN	2	2
Q9NVC6|MED17_HUMAN	2	2
Q6ZTW0|TPGS1_HUMAN	2	2
Q9NZW5|MPP6_HUMAN	2	2
Q9NQY0|BIN3_HUMAN	2	2
Q96T58|MINT_HUMAN	2	2
Q53RE8|ANR39_HUMAN	2	2
Q70EL1|UBP54_HUMAN	2	2
Q96FC9|DDX11_HUMAN	2	2
Q8TED1|GPX8_HUMAN	2	2
Q13144|EI2BE_HUMAN	2	2
Q13445|TMED1_HUMAN	2	2
Q9H290|Q9H290_HUMAN	2	2
Q3ZTR8|Q3ZTR8_HUMAN	2	2
P51452|DUS3_HUMAN	2	2
Q96DV4|RM38_HUMAN	2	2
O15182|CETN3_HUMAN	2	2
Q15070|OXA1L_HUMAN	2	2
Q9UHA3|RLP24_HUMAN	2	2
O95453|PARN_HUMAN	2	2
Q9NSB8|HOME2_HUMAN	2	2
Q8IV63|VRK3_HUMAN	2	2
P63279|UBC9_HUMAN	2	2
P84074|HPCA_HUMAN	2	2
A6PVG9|A6PVG9_HUMAN	2	2
Q9NVR2|INT10_HUMAN	2	2
Q9UBC5|MYO1A_HUMAN	2	2
Q5TC82|RC3H1_HUMAN	2	2
Q08752|PPID_HUMAN	2	2
Q8TEK3|DOT1L_HUMAN	2	2

Q15388|TOM20_HUMAN	2	2
O95801|TTC4_HUMAN	2	2
Q9BQT8|ODC_HUMAN	2	2
Q96E11|RRFM_HUMAN	2	2
P16220|CREB1_HUMAN	2	2
Q86WX3|S19BP_HUMAN	2	2
Q8WXD5|GEMI6_HUMAN	2	2
Q9H8G2|CI082_HUMAN	2	2
Q96LI5|CNO6L_HUMAN	2	2
Q86XZ4|SPAS2_HUMAN	2	2
Q13362|2A5G_HUMAN	2	2
Q8IZP0|ABI1_HUMAN	2	2
Q9H9A6|LRC40_HUMAN	2	2
Q00403|TF2B_HUMAN	2	2
Q9UBD5|ORC3_HUMAN	2	2
P18074|ERCC2_HUMAN	2	2
Q96NL6|SCLT1_HUMAN	2	2
A2RQF4|A2RQF4_HUMAN	2	2
Q96RT1|LAP2_HUMAN	2	2
Q92569|P55G_HUMAN	2	2
Q99543|DNJC2_HUMAN	2	2
Q7Z434|MAVS_HUMAN	2	2
Q9UBS8|RNF14_HUMAN	2	2
Q96SI1|KCD15_HUMAN	2	2
P78312|CD008_HUMAN	2	2
Q96CB8|INT12_HUMAN	2	2
Q9UDY2|ZO2_HUMAN	2	2
Q9Y6I4|UBP3_HUMAN	2	2
Q8IWV7|UBR1_HUMAN	2	2
Q9NVS9|PNPO_HUMAN	2	2
Q9BQ70|TCF25_HUMAN	2	2
Q66LE6|2ABD_HUMAN	2	2
P43378|PTN9_HUMAN	2	2
Q9H7D7|WDR26_HUMAN	2	2
Q9BVS4|RIOK2_HUMAN	2	2
Q9NQS1|AVEN_HUMAN	2	2
Q8IVS2|FABD_HUMAN	2	2
Q9NX04|CA109_HUMAN	2	2
P67812|SC11A_HUMAN	2	2
Q96CB9|NSUN4_HUMAN	2	2
Q86UU1|PHLB1_HUMAN	2	2
Q9BYK8|PR285_HUMAN	2	2
A7MCZ2|A7MCZ2_HUMAN	2	2
B2R5X0|B2R5X0_HUMAN	2	2
Q8IZV5|RDH10_HUMAN	2	2
Q6RFH5|WDR74_HUMAN	2	2
Q9BSK2|S2533_HUMAN	2	2

Q96NW4|ANR27_HUMAN	2	2
Q86YW9|MD12L_HUMAN	2	2
Q6N021|TET2_HUMAN	2	2
Q9UL03|INT6_HUMAN	2	2
Q9Y243|AKT3_HUMAN	2	2
Q5HYK3|COQ5_HUMAN	2	2
Q9BVS5|TRMI_HUMAN	2	2
O43353|RIPK2_HUMAN	2	2
Q9UNX3|RL26L_HUMAN	2	2
Q96CG3|TIFA_HUMAN	2	2
Q9BUH6|CI142_HUMAN	2	2
Q8NI37|PPTC7_HUMAN	2	2
Q9HD42|CHM1A_HUMAN	2	2
Q13112|CAF1B_HUMAN	2	2
Q9H4L7|SMRCD_HUMAN	2	2
