# Supplementary figures and images for: Proteomic Analysis Shows Synthetic Oleanane Triterpenoid Binds to mTOR
Source: PLoS One. 2011 Jul 27;6(7):e22862. doi: 10.1371/journal.pone.0022862 (PMC3144948; doi:10.1371/journal.pone.0022862)

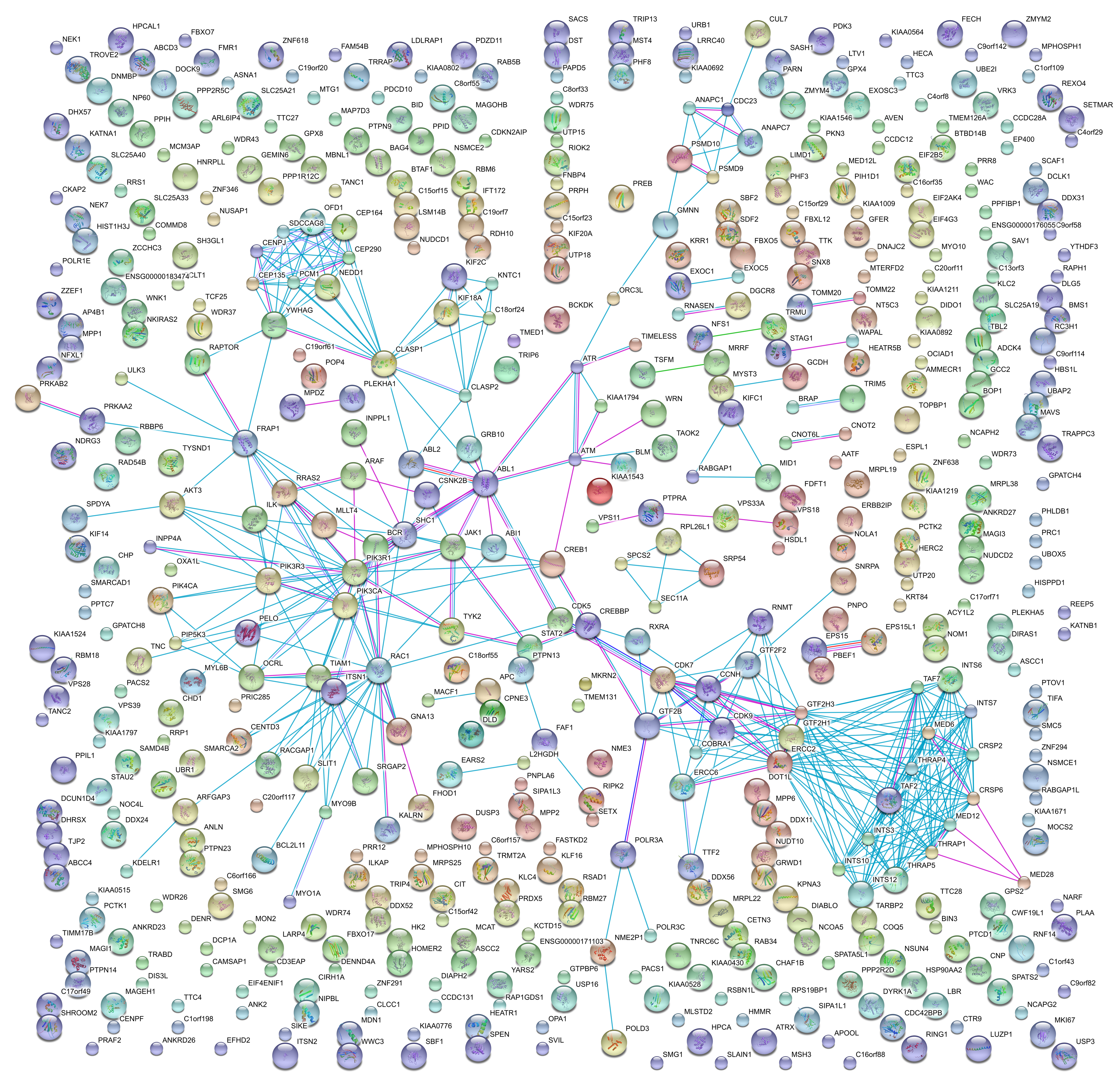

Supplement: Figure S2 — High resolution, scalable image of the network map depicted in figure 3c. (TIF) [file pone.0022862.s002.tif]

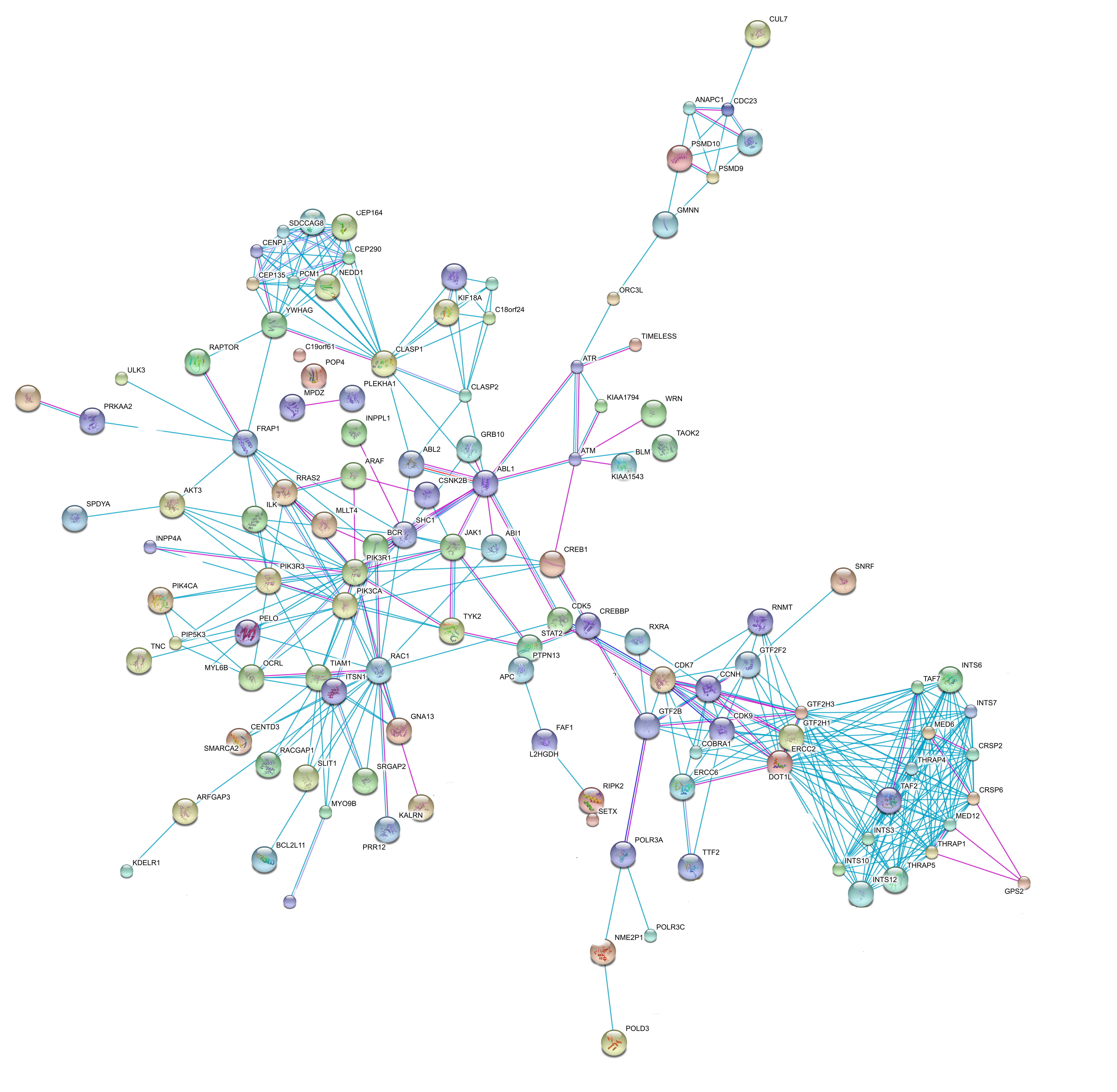

Supplement: Figure S3 — High resolution, scalable image of the sub-network map depicted in figure 3d. (TIF) [file pone.0022862.s003.tif]
